# *fac*-Tri­aqua­(1,10-phenanthroline-κ^2^ *N*,*N*′)(sulfato-κ*O*)cobalt(II): crystal structure, Hirshfeld surface analysis and computational study

**DOI:** 10.1107/S2056989020006271

**Published:** 2020-05-15

**Authors:** Zouaoui Setifi, Huey Chong Kwong, Edward R. T. Tiekink, Thierry Maris, Fatima Setifi

**Affiliations:** a Département de Technologie, Faculté de Technologie, Université 20 Août 1955-Skikda, BP 26, Route d’El-Hadaiek, Skikda 21000, Algeria; b Laboratoire de Chimie, Ingénierie Moléculaire et Nanostructures (LCIMN), Université Ferhat Abbas Sétif 1, Sétif 19000, Algeria; c Research Centre for Crystalline Materials, School of Science and Technology, Sunway University, 47500 Bandar Sunway, Selangor Darul Ehsan, Malaysia; d Department of Chemistry, Université de Montréal, 2900 Edouard-Montpetit Blvd, Montreal, Quebec, H3T1J4, Canada

**Keywords:** crystal structure, cobalt(II), hydrogen bonding, Hirshfeld surface analysis

## Abstract

The Co^II^ atom in the title complex, [Co(SO_4_)(C_12_H_8_N_2_)(H_2_O)_3_] (or C_12_H_14_CoN_2_O_7_S), is octa­hedrally coordinated within a cis-N_2_O_4_ donor set defined by the chelating N-donors of the 1,10-phenanthroline ligand, sulfate-O and three aqua-O atoms, the latter occupying an octa­hedral face. In the crystal, supra­molecular layers lying parallel to (110) are sustained by aqua-O—H⋯O(sulfate) hydrogen bonding. The layers stack along the *c*-axis direction with the closest directional inter­action between them being a weak phenanthroline-C—H⋯O(sulfate) contact. There are four significant types of contact contributing to the calculated Hirshfeld surface: at 44.5%, the major contribution comes from O—H⋯O contacts followed by H⋯H (28.6%), H⋯C/C⋯H (19.5%) and C⋯C (5.7%) contacts. The dominance of the electrostatic potential force in the mol­ecular packing is also evident in the calculated energy frameworks. The title complex is isostructural with its manganese, zinc and cadmium containing analogues and isomeric with its *mer*-tri­aqua analogue.

## Abstract 2

The Co^II^ atom in the title complex is octa­hedrally coordinated within an N_2_O_4_ donor set defined by two N-atom donors of the 1,10-phenanthroline ligand, sulfate-O and three aqua-O atoms, the latter occupying an octa­hedral face. In the crystal, supra­molecular layers parallel to (110) are sustained by aqua-O—H⋯O(sulfate) hydrogen bonding.

## Chemical context   

As a consequence of their ability to link metal ions in a variety of different ways, polynitrile anions, either functioning alone or in combination with neutral co-ligands, provide opportunities for the generation of mol­ecular architectures with varying dimensions and topologies (Benmansour *et al.*, 2012 ▸). The presence of other potential donor groups such as those derived from –OH, –SH or –NH_2_, together with their rigidity and electronic delocalization, mean that polynitrile anions can also lead to new magnetic and luminescent coordination polymers based on transition-metal ions (Benmansour *et al.*, 2010 ▸; Kayukov *et al.*, 2017 ▸; Lehchili *et al.*, 2017 ▸; Setifi *et al.*, 2017 ▸). Furthermore, the use of polynitrile anions for the synthesis of inter­esting discrete and polymeric bis­table materials has been described (Setifi *et al.*, 2014 ▸; Milin *et al.*, 2016 ▸; Pittala *et al.*, 2017 ▸). In view of this coordinating ability, these ligands have also been explored for their utility in developing materials capable of magnetic exchange coupling (Addala *et al.*, 2015 ▸; Déniel *et al.*, 2017 ▸). It was during the course of attempts to prepare such complexes with 1,10-phenanthroline as a co-ligand that the title complex, (I), was unexpectedly obtained. Herein, the crystal and mol­ecular structures of (I) are described, a study complemented by an analysis of the mol­ecular packing by calculating the Hirshfeld surfaces as well as a computational chemistry study.
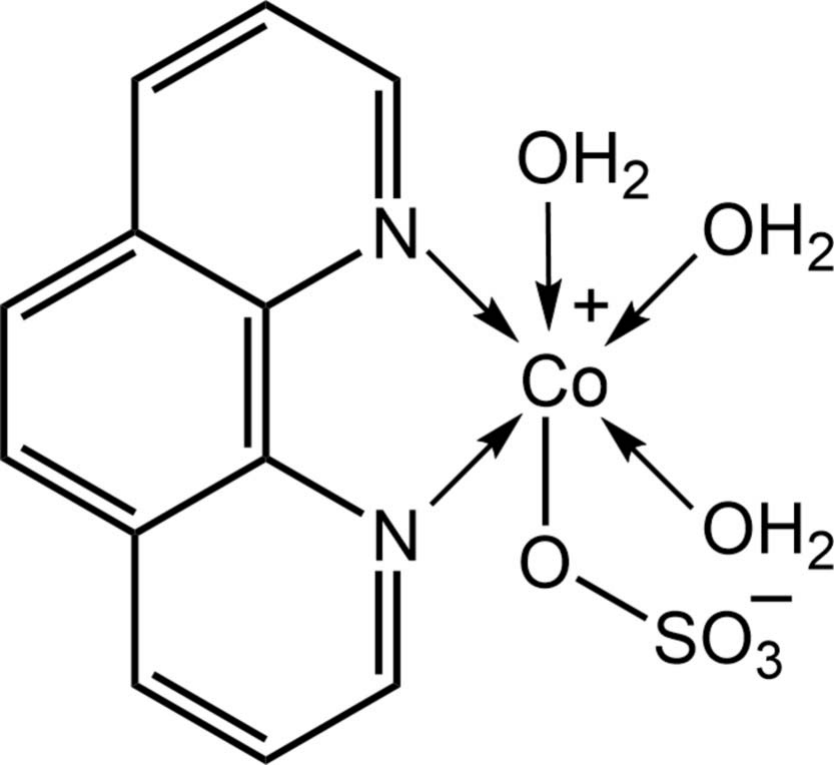


## Structural commentary   

**Figure 1:**
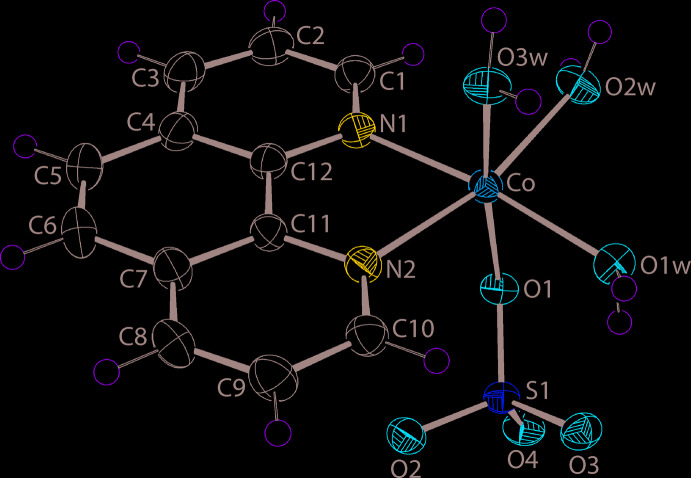
The mol­ecular structure of (I) showing the atom-labelling scheme and displacement ellipsoids at the 50% probability level.

The mol­ecule of (I) is shown in Fig. 1 ▸ and selected geometric parameters are collated in Table 1 ▸. The Co^II^ complex features a chelating 1,10-phenanthroline ligand, a monodentate sulfate di-anion and three coordinated water mol­ecules. The resulting N_2_O_4_ donor set defines a distorted octa­hedral coordination geometry for the Co^II^ atom, with the water mol­ecules occupying one octa­hedral face. The greatest deviations from a regular geometry is seen in the restricted bite angle subtended by the 1,10-phenanthroline ligand, *i.e*. N1—Co1—N2 = 78.21 (6)°, and in the *trans* O2*W*—Co—N2 angle of 166.55 (6)°. The Co—N bond lengths are equal within experimental error but the Co—O(aqua) bonds span an experimentally distinct range, Table 1 ▸. The observation that the shortest and longest Co—O(aqua) bonds have each aqua-O atom *trans* to a nitro­gen atom suggests the differences in bond lengths are due to the considerable hydrogen bonding operating in the crystal. Indeed, there is an intra­molecular aqua-O1*W*—H⋯O3(sulfate) hydrogen bond, Table 2 ▸. The coordinated sulfate-O1 atom forms the longer of the four sulfate-S—O bonds, Table 1 ▸. The S—O bond lengths formed by the non-coordinating sulfate-oxygen atoms spans an experimentally distinct range of 1.4616 (14) Å for S1—O2, to 1.4813 (14) Å for S1—O3. As discussed below, the sulfate-O1–O4 oxygen atoms form, respectively, one, one, two and two hydrogen bonds with the water mol­ecules, which is consistent with the S1—O2 bond length being the shortest of the four bonds. The above notwithstanding, it is likely that the formal negative charge on the SO_3_ residue is delocalized over the three non-coordinating S—O bonds.

## Supra­molecular features   

**Figure 2:**
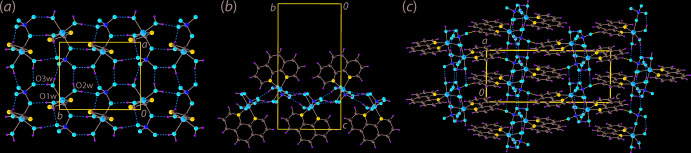
Mol­ecular packing in the crystal of (I): (*a*) supra­molecular layer sustained by aqua-O—H⋯O(sulfate) hydrogen bonding shown as orange dashed lines, only the five-membered chelate rings are shown for reasons of clarity, (*b*) a side-on view of the layer shown in (*a*) and (*c*) a view of the unit-cell contents down the *b* axis showing the stacking of layers along the *c*-axis direction, with the phenanthroline-C—H⋯O(sulfate) inter­actions between layers shown as blue dashed lines.

Each of the aqua ligands donates two hydrogen bonds to different sulfate-O atoms, one of these hydrogen bonds is intra­molecular while the remaining are inter­molecular, Table 2 ▸. The result of the hydrogen bonding is the formation of a supra­molecular layer lying parallel to (110). A simplified view of the hydrogen bonding scheme is shown in Fig. 2 ▸(*a*). The aqua mol­ecule forming the intra­molecular O1*W*—H⋯O3 hydrogen bond forms a second hydrogen bond to the coordinated O1 atom of a symmetry-related mol­ecule, and the O2*W* aqua ligand of this mol­ecule connects to the O3 atom of the original mol­ecule, leading to the formation of a non-symmetric eight-membered {⋯HOH⋯O⋯HOCoO} synthon. The second hydrogen atom of the O2*W* ligand forms a connection to a sulfate-O4 atom, which is also hydrogen bonded to an O3*W* mol­ecule, which forms an additional link to a symmetry related sulfate-O2 atom with the result a {⋯HOH⋯OSO⋯HOH⋯O} non-symmetric ten-membered synthon is formed. Two additional eight-membered synthons, {HOCoOH⋯OSO}, are formed as a result of the hydrogen-bonding scheme as adjacent pairs of aqua mol­ecules effectively bridge two sulfoxide residues. As seen from Fig. 2 ▸(*b*), the 1,10-phenanthroline mol­ecules project to either side of the supra­molecular layer. The layers inter-digitate along [001], Fig. 2 ▸(*c*), with the closest connections between layers being phenanthroline-C—H⋯O2(sulfate) inter­actions, Table 2 ▸. A deeper analysis of the mol­ecular packing is found in the next two sections of this paper.

## Hirshfeld surface analysis   

**Figure 3:**
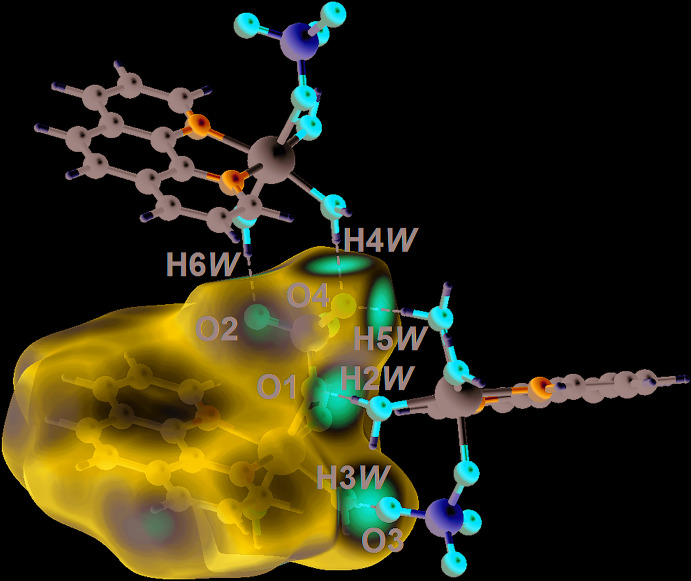
A view of the Hirshfeld surface mapped over *d*_norm_ for (I) in the range of −0.729 to +1.105 arbitrary units, highlighting O—H⋯O inter­actions.

**Figure 4:**
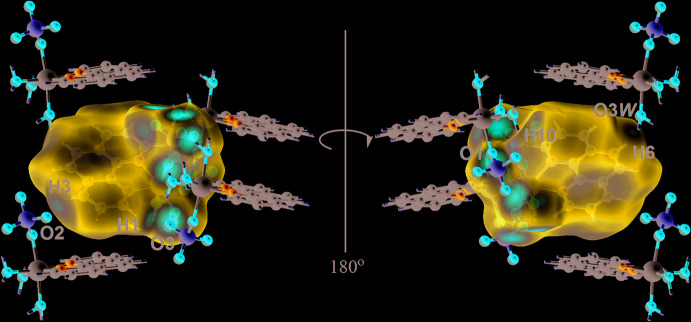
Two views of the Hirshfeld surface mapped over *d*_norm_ for (I) in the range of −0.729 to +1.105 arbitrary units, highlighting weak C—H⋯O inter­actions and short contacts.

**Figure 5:**
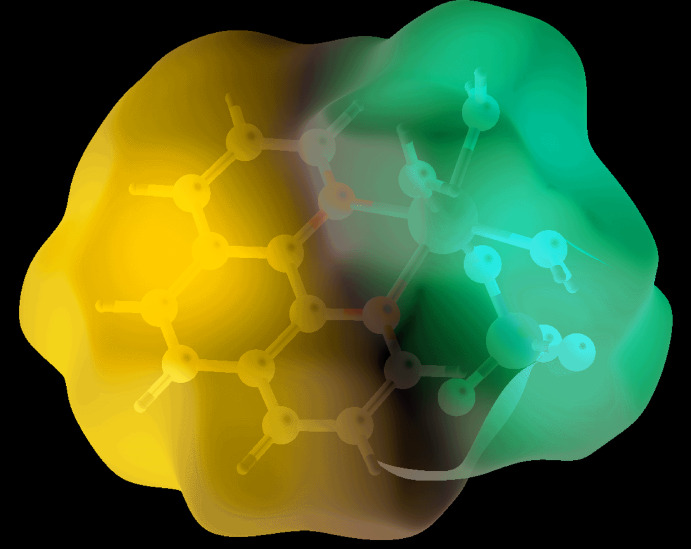
A view of the Hirshfeld surface mapped over the calculated electrostatic potential for (I). The potentials were calculated using the STO-3G basis set at Hartree–Fock level of theory over a range of −4.381 to 4.109 atomic units. The red and blue regions represent negative and positive electrostatic potentials, respectively.

In order to understand further the inter­actions operating in the crystal of (I), the Hirshfeld surfaces and two-dimensional fingerprint plots were calculated employing the program *Crystal Explorer 17* (Turner *et al.*, 2017 ▸) and literature procedures (Tan *et al.*, 2019 ▸). The inter­molecular O—H⋯O hydrogen bonds in (I), Table 2 ▸, are characterized as pairs of bright-red spots near the aqua-O and sulfate-O atoms on the Hirshfeld surface mapped over *d*_norm_ shown in Fig. 3 ▸. The faint-red spots near the phenanthroline-C—H (H1, H3 H6 and H10) atoms on the *d*_norm_-mapped Hirshfeld surface in the two views of Fig. 4 ▸ represent the influence of the weak C3—H3⋯O2 and C10—H10⋯O1 inter­actions as well as H1⋯O3, H6⋯O3*W* short contacts, Table 3 ▸. The donors and acceptors of the weak C—H⋯O inter­action are viewed as blue and red regions on the Hirshfeld surface mapped over the calculated electrostatic potential in Fig. 5 ▸, and which correspond to positive and negative electrostatic potentials.

**Figure 6:**
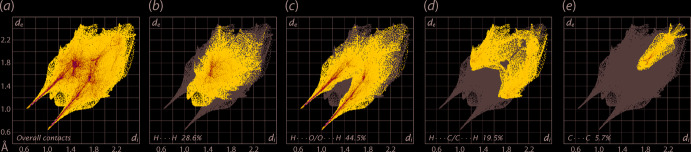
(*a*) The overall two-dimensional fingerprint plots for (I), and those delineated into (*b*) H⋯H, (*c*) O⋯H/H⋯O, (*d*) C⋯H/H⋯C and (*e*) C⋯C contacts.

The overall two-dimensional fingerprint plot of (I) is shown in Fig. 6 ▸(*a*). The overall contacts are also delineated into H⋯H, H⋯O/O⋯H, H⋯C/C⋯H and C⋯C contacts, as displayed in Fig. 6 ▸(*b*)–(*e*), respectively. The short inter­atomic H⋯H contacts are characterized as the pair of beak-shaped tips at *d*_e_ + *d*_i_ ∼2.3 Å, Fig. 6 ▸(*b*), and contribute 28.6% to the overall surface contacts. The significant O—H⋯O contacts between the aqua- and sulfate-O atoms make the major contribution to the overall contacts (44.5%), and these are represented as pairs of well-defined spikes at *d*_e_ + *d*_i_ ∼1.7 Å in Fig. 6 ▸(*c*). The short inter­atomic H⋯C/C⋯H (19.5%) and C⋯C (5.7%) contacts are, respectively, characterized as pairs of broad symmetrical wings at *d*_e_ + *d*_i_ ∼2.9 Å in Fig. 6 ▸(*d*), and the vase-shaped distribution of points at *d*_e_ + *d*_i_ ∼3.5 Å in Fig. 6 ▸(*e*). The accumulated contribution of the remaining inter­atomic contacts is less than 2% and has a negligible effect on the packing.

## Computational chemistry   

**Figure 7:**
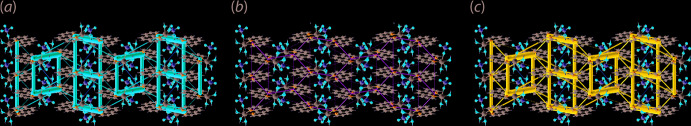
Perspective views of the energy frameworks calculated for (I), showing the (*a*) electrostatic potential force, (*b*) dispersion force and (*c*) total energy, each plotted down the *b* axis. The radii of the cylinders are proportional to the relative magnitudes of the corresponding energies and were adjusted to the same scale factor of 20 with a cut-off value of 5 kJ mol^−1^ within 2 × 2 × 2 unit cells.

In the present analysis, the pairwise inter­action energies between the mol­ecules in the crystal were calculated by summing up four different energy components, *i.e*. the electrostatic (*E*_ele_), polarization (*E*_pol_), dispersion (*E*_dis_) and exchange-repulsion (*E*_rep_) energy terms, after Turner *et al.* (2017 ▸). These energies were obtained by applying the wave functions calculated at the B3LYP/6-31G(d,p) level of theory. The benchmarked energies were scaled according to Mackenzie *et al.* (2017 ▸) while *E*_ele_, *E*_pol_, *E*_dis_ and *E*_rep_ were scaled as 1.057, 0.740, 0.871 and 0.618, respectively (Edwards *et al.*, 2017 ▸). The inter­molecular inter­action energies are collated in Table 4 ▸. Consistent with the presence of strong O—H⋯O hydrogen-bonding inter­actions in the crystal, the electrostatic energy component has a major influence in the formation of supra­molecular architecture of (I), Table 4 ▸. The energy associated with the C—H⋯O inter­actions involving the sulfate-O atoms (−66.8 and −55.7 kJ mol^−1^) are greater than for the C—H⋯O inter­action involving the aqua-O atoms (−30.6 kJ mol^−1^). The energy frameworks were also computed and illustrate the above conclusions, Fig. 7 ▸. These clearly demonstrate the dominance of the electrostatic potential energy in the mol­ecular packing.

## Database survey   

There are several literature analogues of (I), *i.e*. mol­ecules conforming to the general formula *fac*-*M*(1,10-phenanthroline)(OH_2_)_3_OSO_3_. These include *M* = Mn (XATNAH; Zheng *et al.*, 2000 ▸), *M* = Zn (IJOQAA; Liu *et al.*, 2011 ▸) and *M* = Cd (RACWUO; Li *et al.*, 2003 ▸). The three literature structures are isostructural with (I). Literature analogues are also available for the isomeric *mer*-*M*(1,10-phenanthroline)(OH_2_)_3_OSO_3_ species, *i.e. M* = Mn (UGOJUV; Zheng *et al.*, 2002 ▸), *M* = Fe (MIKJAS; Li *et al.*, 2007 ▸), *M* = Co (FICNOU; Li & Zhou, 1987 ▸) and *M* = Ni (ESUZOH; He *et al.*, 2003 ▸). The four *mer*-isomers are also isostructural, crystallizing in the monoclinic space group *P*2_1_/*c*. There are two pairs of structures (containing Mn and Co) crystallizing in both forms. For the Mn complexes, the authors reporting the structure of the *mer*-isomer indicated that both forms were formed concomitantly from the slow evaporation of a methanol solution of the complex (Zheng *et al.*, 2002 ▸). To a first approximation, the mol­ecular packing in the *mer* form resembles that for the *fac*-isomer in that supra­molecular layers are formed by hydrogen bonding whereby each aqua ligand hydrogen bonds to two different sulfate-O atoms, *i.e*. as for (I).

The key difference in the packing between the two isomers arises as one sulfate-O atom in the *mer*-isomer participates in three hydrogen bonds at the expense of the hydrogen bond involving the coordinated sulfate-O1 atom. The presence of inter-layer phenanthroline-C—H⋯O(sulfate) inter­actions persist as for the *fa*c-isomer with the crucial difference that π–π stacking inter­actions are evident in the inter-layer region of the *mer*-form with the shortest separation being 3.76 Å.

The different packing arrangements result in different densities with that for (I) of 1.776 g cm^−3^ being greater than 1.723 g cm^−3^ for the *mer*-isomer (FICNOU; Li & Zhou, 1987 ▸). The calculated packing efficiencies follow this trend being 72.8 and 66.5%, respectively. Similar results are noted for the pair of Mn structures, *i.e*. 1.690 g cm^−3^ and 71.1% for the *fac*-isomer (Zheng *et al.*, 2000 ▸) *c.f.* 1.643 g cm^−3^ and 68.7% for the *mer*-isomer (Zheng *et al.*, 2000 ▸). The consistency of these parameters may suggest that the *fac*-isomer in these *M*(1,10-phenanthroline)(OH_2_)_3_OSO_3_ complexes is the thermodynamically more stable form.

Given the isostructural relationship in the series (I), IJOQAA, RACWUO and XATNAH, it was thought of inter­est to compare the percentage contributions of the difference inter­molecular contacts to the calculated Hirshfeld surfaces. Thus, these were calculated for the three literature structures as were the overall and delineated two-dimensional fingerprint plots. Qualitatively, the fingerprint plots had the same general appearance in accord with expectation (Jotani *et al.*, 2019 ▸). The calculated percentage contributions to the Hirshfeld surfaces for the four complexes are collated in Table 5 ▸. Clearly and as would be expected, the data in Table 5 ▸ reveal a high degree of concordance in the percentage contributions to the Hirshfeld surfaces between the four isostructural complexes.

## Synthesis and crystallization   

The title compound was synthesized solvothermally under autogenous pressure from a mixture of CoSO_4_·7H_2_O (28 mg, 0.1 mmol), 1,10-phenanthroline (18 mg, 0.1 mmol) and K(tcnoet) (45 mg, 0.2 mmol) in water–methanol (4:1*v*/*v*, 25 ml); where tcnoet is 1,1,3,3-tetra­cyano-2-eth­oxy­propenide. The mixture was sealed in a Teflon-lined autoclave and held at 403 K for 2 days, and then cooled to room temperature at a rate of 10 K h^−1^; yield: 35%. Light-pink blocks of the title complex suitable for single-crystal X-ray diffraction were selected directly from the synthesized product.

## Refinement   

Crystal data, data collection and structure refinement details are summarized in Table 6 ▸. The carbon-bound H atoms were placed in calculated positions (C—H = 0.95 Å) and were included in the refinement in the riding-model approximation, with *U*_iso_(H) set to 1.2*U*_eq_(C). The oxygen-bound H atoms were located from a difference-Fourier map and refined with O—H = 0.84±0.01 Å, and with *U*_iso_(H) set to 1.5*U*_eq_(O). Owing to poor agreement, four reflections, *i.e*. (0 1 4), (0 0 2), (0 1 2) and (0 0 4), were omitted from the final cycles of refinement. The absolute structure was determined based on differences in Friedel pairs included in the data set.

## Supplementary Material

Crystal structure: contains datablock(s) I, global. DOI: 10.1107/S2056989020006271/hb7911sup1.cif

Structure factors: contains datablock(s) I. DOI: 10.1107/S2056989020006271/hb7911Isup2.hkl

CCDC reference: 2002737

Additional supporting information:  crystallographic information; 3D view; checkCIF report

## Figures and Tables

**Table 1:** Selected bond lengths (Å)

Co—O1	2.1386 (13)	Co—N2	2.1453 (16)
Co—O1*W*	2.1110 (14)	S1—O1	1.4997 (13)
Co—O2*W*	2.0782 (15)	S1—O2	1.4616 (14)
Co—O3*W*	2.1024 (14)	S1—O3	1.4813 (14)
Co—N1	2.1356 (15)	S1—O4	1.4784 (14)

**Table 2:** Hydrogen-bond geometry (Å, °)

*D*—H⋯*A*	*D*—H	H⋯*A*	*D*⋯*A*	*D*—H⋯*A*
O1*W*—H1*W*⋯O3	0.84 (2)	1.89 (2)	2.680 (2)	158 (2)
O1*W*—H2*W*⋯O1^i^	0.83 (1)	1.95 (1)	2.7818 (19)	172 (2)
O2*W*—H3*W*⋯O3^ii^	0.84 (2)	1.91 (2)	2.744 (2)	175 (3)
O2*W*—H4*W*⋯O4^iii^	0.85 (1)	1.93 (1)	2.770 (2)	167 (3)
O3*W*—H5*W*⋯O4^i^	0.82 (2)	1.95 (2)	2.7548 (19)	168 (3)
O3*W*—H6*W*⋯O2^iii^	0.82 (2)	1.84 (2)	2.6560 (19)	178 (3)
C3—H3⋯O2^iv^	0.95	2.45	3.252 (3)	142

Symmetry codes: (i) 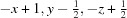; (ii) 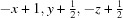; (iii) 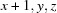; (iv) 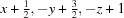.

**Table 3:** A summary of short inter­atomic contacts (Å) in (I)*^*a*^*

Contact	Distance	Symmetry operation
H2*W*⋯O1*^*b*^*	1.81	−*x* + 1, *y* − 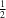 , −*z* + 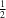
H3*W*⋯O3*^*b*^*	1.76	−*x* + 1, *y* + 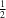 , −*z* + 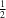
H4*W*⋯O4*^*b*^*	1.81	*x* + 1, *y*, *z*
H5*W*⋯O4*^*b*^*	1.79	−*x* + 1, *y* − 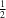 , −*z* + 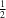
H6*W*⋯O2*^*b*^*	1.67	*x* + 1, *y*, *z*
H1⋯O3	2.33	−*x* + 1, *y* + 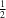 , −*z* + 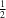
H3⋯O2	2.35	*x* + 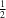 , −*y* + 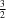 , − *z* + 1
H6⋯O3*W*	2.51	*x* − 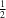 , −*y* + 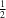 , − *z* + 1
H10⋯O1	2.40	−*x* + 1, *y* − 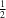 , − *z* + 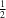

Notes: (*a*) The inter­atomic distances are calculated in *Crystal Explorer 17* (Turner *et al.*, 2017 ▸) whereby the *X*—H bond lengths are adjusted to their neutron values; (*b*) these inter­actions correspond to conventional hydrogen bonds.

**Table 4:** A summary of inter­action energies (kJ mol^−1^) calculated for (I)

Contact	*R* (Å)	*E*_ele_	*E*_pol_	*E*_dis_	*E*_rep_	*E*_tot_
O1*W*—H2*W*⋯O1^i^ +	6.78	−330.8	−116.8	−49.6	180.1	−368.1
O3*W*—H5*W*⋯O4^i^ +						
O2*W*—H3*W*⋯O3^ii^ +						
C10—H10⋯O1^i^						
O3*W*—H6*W*⋯O2^iii^ +	7.97	−198.3	−63.8	−16.4	121.0	−196.4
O2*W*—H4*W*⋯O4^iii^						
C5—H5⋯O3^v^ +	10.47	−46.2	−19.3	−9.8	7.8	−66.8
C6—H6⋯O4^v^						
C3—H3⋯O2^iv^	7.64	−17.3	−30.2	−42.3	35.3	−55.7
C6—H6⋯O3*W* ^vi^	8.03	−2.3	−13.7	−37.7	24.0	−30.6

Symmetry operations: (i) −*x* + 1, *y* − 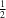, −*z* + 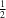; (ii) − *x* + 1, *y* + 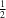, − *z* + 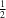; (iii) *x* + 1, *y*, *z*; (iv) *x* + 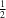, −*y* + 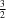, −*z* + 1; (v) −*x* + 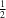, − *y* + 1, *z* + 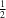; (vi) *x* – 1/2, −*y* + 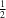, −*z* + 1.

**Table 5:** Percentage contributions to inter­molecular contacts on the Hirshfeld surface calculated for (I)

Contact		Percentage contribution		
	(I), *M* = Co	IJOQAA, *M* = Zn	RACWUO, *M* = Cd	XATNAH, *M* = Mn
H⋯H	28.6	30.1	27.6	27.2
H⋯O/O⋯H	44.5	43.3	45.8	45.9
H⋯C/C⋯H	19.5	19.1	19.2	19.1
C⋯C	5.7	5.7	5.2	5.6
Others	1.7	1.8	2.2	2.2

**Table 6:** Experimental details

Crystal data	
Chemical formula	[Co(SO_4_)(C_12_H_8_N_2_)(H_2_O)_3_]
*M*_r_	389.24
Crystal system, space group	Orthorhombic, *P*2_1_2_1_2_1_
Temperature (K)	150
*a*, *b*, *c* (Å)	7.9732 (4), 9.5589 (4), 19.0955 (9)
*V* (Å^3^)	1455.36 (12)
*Z*	4
Radiation type	Ga *K*α, λ = 1.34139 Å
μ (mm^−1^)	7.61
Crystal size (mm)	0.08 × 0.08 × 0.05

Data collection	
Diffractometer	Bruker Venture Metaljet
Absorption correction	Multi-scan (*SADABS*; Bruker, 2016 ▸)
*T*_min_, *T*_max_	0.064, 0.155
No. of measured, independent and observed [*I* > 2σ(*I*)] reflections	25223, 3202, 3126
*R*_int_	0.033
(sin θ/λ)_max_ (Å^−1^)	0.650

Refinement	
*R*[*F* ^2^ > 2σ(*F* ^2^)], *wR*(*F* ^2^), *S*	0.017, 0.046, 0.99
No. of reflections	3202
No. of parameters	227
No. of restraints	6
H-atom treatment	H atoms treated by a mixture of independent and constrained refinement
Δρ_max_, Δρ_min_ (e Å^−3^)	0.51, −0.58
Absolute structure	Flack *x* determined using 1194 quotients [(*I* ^+^)−(*I* ^−^)]/[(*I* ^+^)+(*I* ^−^)] (Parsons *et al.*, 2013 ▸).
Absolute structure parameter	0.0101 (17)

Computer programs: *APEX2* and *SAINT* (Bruker, 2013 ▸), *SHELXS* (Sheldrick, 2015*a* ▸), *SHELXL2018/3* (Sheldrick, 2015*b* ▸), *ORTEP-3 for Windows* (Farrugia, 2012 ▸), *DIAMOND* (Brandenburg, 2006 ▸) and *publCIF* (Westrip, 2010 ▸).

## supplementary crystallographic information

### Crystal data

**Table:**

[Co(SO_4_)(C_12_H_8_N_2_)(H_2_O)_3_]	*D*_x_ = 1.776 Mg m^−^^3^
*M**_r_* = 389.24	Ga *K*α radiation, λ = 1.34139 Å
Orthorhombic, *P*2_1_2_1_2_1_	Cell parameters from 9840 reflections
*a* = 7.9732 (4) Å	θ = 4.0–60.7°
*b* = 9.5589 (4) Å	µ = 7.61 mm^−^^1^
*c* = 19.0955 (9) Å	*T* = 150 K
*V* = 1455.36 (12) Å^3^	Prism, light-pink
*Z* = 4	0.08 × 0.08 × 0.05 mm
*F*(000) = 796	

### Data collection

**Table:**

Bruker Venture Metaljet diffractometer	3202 independent reflections
Radiation source: Metal Jet, Gallium Liquid Metal Jet Source	3126 reflections with *I* > 2σ(*I*)
Helios MX Mirror Optics monochromator	*R*_int_ = 0.033
Detector resolution: 10.24 pixels mm^-1^	θ_max_ = 60.6°, θ_min_ = 4.5°
ω and φ scans	*h* = −10→10
Absorption correction: multi-scan (SADABS; Bruker, 2016)	*k* = −12→12
*T*_min_ = 0.064, *T*_max_ = 0.155	*l* = −24→24
25223 measured reflections	

### Refinement

**Table:**

Refinement on *F*^2^	Hydrogen site location: mixed
Least-squares matrix: full	H atoms treated by a mixture of independent and constrained refinement
*R*[*F*^2^ > 2σ(*F*^2^)] = 0.017	*w* = 1/[σ^2^(*F*_o_^2^) + (0.0202*P*)^2^] where *P* = (*F*_o_^2^ + 2*F*_c_^2^)/3
*wR*(*F*^2^) = 0.046	(Δ/σ)_max_ = 0.001
*S* = 0.99	Δρ_max_ = 0.51 e Å^−^^3^
3202 reflections	Δρ_min_ = −0.58 e Å^−^^3^
227 parameters	Extinction correction: SHELXL-2018/3 (Sheldrick, 2015*b*), Fc^*^=kFc[1+0.001xFc^2^λ^3^/sin(2θ)]^-1/4^
6 restraints	Extinction coefficient: 0.0057 (5)
Primary atom site location: structure-invariant direct methods	Absolute structure: Flack *x* determined using 1194 quotients [(*I*^+^)-(*I*^-^)]/[(*I*^+^)+(*I*^-^)] (Parsons *et al.*, 2013).
Secondary atom site location: difference Fourier map	Absolute structure parameter: 0.0101 (17)

### Special details

**Table:**

Geometry. All esds (except the esd in the dihedral angle between two l.s. planes) are estimated using the full covariance matrix. The cell esds are taken into account individually in the estimation of esds in distances, angles and torsion angles; correlations between esds in cell parameters are only used when they are defined by crystal symmetry. An approximate (isotropic) treatment of cell esds is used for estimating esds involving l.s. planes.

### Fractional atomic coordinates and isotropic or equivalent isotropic displacement parameters (Å^2^)

**Table:**

	*x*	*y*	*z*	*U*_iso_*/*U*_eq_	
Co	0.63811 (3)	0.53373 (3)	0.31720 (2)	0.02111 (9)	
S1	0.24258 (5)	0.56910 (4)	0.27630 (2)	0.02212 (11)	
O1	0.40704 (16)	0.63702 (14)	0.29351 (7)	0.0239 (3)	
O2	0.17822 (17)	0.49545 (15)	0.33775 (7)	0.0291 (3)	
O3	0.26932 (17)	0.46986 (15)	0.21777 (7)	0.0289 (3)	
O4	0.12543 (18)	0.68089 (14)	0.25463 (7)	0.0290 (3)	
O1W	0.60122 (18)	0.42633 (14)	0.22184 (7)	0.0267 (3)	
H1W	0.5005 (18)	0.431 (3)	0.2097 (13)	0.040*	
H2W	0.607 (3)	0.3405 (14)	0.2144 (14)	0.040*	
O2W	0.77952 (18)	0.68717 (15)	0.26762 (8)	0.0296 (3)	
H3W	0.768 (4)	0.7735 (15)	0.2743 (14)	0.044*	
H4W	0.8839 (17)	0.673 (3)	0.2607 (15)	0.044*	
O3W	0.85965 (17)	0.41650 (15)	0.32821 (7)	0.0281 (3)	
H5W	0.874 (4)	0.353 (2)	0.2999 (12)	0.042*	
H6W	0.9583 (19)	0.440 (3)	0.3324 (14)	0.042*	
N1	0.6414 (2)	0.64091 (16)	0.41532 (8)	0.0252 (3)	
N2	0.5377 (2)	0.37822 (16)	0.38651 (8)	0.0240 (3)	
C1	0.6826 (3)	0.7733 (2)	0.42840 (11)	0.0307 (4)	
H1	0.716162	0.831005	0.390391	0.037*	
C2	0.6788 (3)	0.8312 (2)	0.49569 (12)	0.0349 (5)	
H2	0.706310	0.926898	0.502638	0.042*	
C3	0.6351 (3)	0.7487 (2)	0.55149 (12)	0.0357 (5)	
H3	0.634774	0.786001	0.597609	0.043*	
C4	0.5906 (3)	0.6081 (2)	0.53970 (11)	0.0311 (4)	
C5	0.5383 (3)	0.5154 (3)	0.59456 (11)	0.0374 (5)	
H5	0.537335	0.547683	0.641615	0.045*	
C6	0.4904 (3)	0.3828 (3)	0.58034 (11)	0.0394 (5)	
H6	0.456658	0.323326	0.617613	0.047*	
C7	0.4897 (3)	0.3302 (2)	0.50992 (11)	0.0315 (4)	
C8	0.4422 (3)	0.1926 (2)	0.49259 (12)	0.0358 (5)	
H8	0.410012	0.128509	0.528178	0.043*	
C9	0.4430 (3)	0.1523 (2)	0.42371 (12)	0.0350 (5)	
H9	0.411464	0.059775	0.411121	0.042*	
C10	0.4904 (3)	0.2480 (2)	0.37212 (11)	0.0290 (4)	
H10	0.488688	0.218795	0.324580	0.035*	
C11	0.5388 (2)	0.4191 (2)	0.45479 (10)	0.0251 (4)	
C12	0.5919 (2)	0.5600 (2)	0.46986 (10)	0.0258 (4)	

### Atomic displacement parameters (Å^2^)

**Table:**

	*U*^11^	*U*^22^	*U*^33^	*U*^12^	*U*^13^	*U*^23^
Co	0.02084 (13)	0.02088 (13)	0.02160 (13)	−0.00016 (10)	0.00033 (10)	0.00127 (10)
S1	0.0203 (2)	0.0203 (2)	0.0258 (2)	−0.00016 (15)	−0.00058 (17)	−0.00105 (15)
O1	0.0203 (6)	0.0225 (6)	0.0291 (6)	−0.0006 (5)	−0.0005 (5)	−0.0017 (5)
O2	0.0262 (6)	0.0301 (7)	0.0311 (7)	−0.0029 (6)	0.0008 (5)	0.0037 (5)
O3	0.0282 (6)	0.0279 (6)	0.0306 (7)	−0.0006 (6)	−0.0015 (6)	−0.0074 (6)
O4	0.0235 (6)	0.0256 (6)	0.0378 (7)	0.0016 (6)	−0.0023 (6)	0.0027 (5)
O1W	0.0273 (7)	0.0240 (6)	0.0287 (7)	0.0042 (5)	−0.0013 (6)	−0.0016 (5)
O2W	0.0244 (7)	0.0244 (6)	0.0399 (8)	0.0008 (6)	0.0046 (6)	0.0047 (6)
O3W	0.0221 (6)	0.0250 (6)	0.0371 (7)	0.0012 (6)	−0.0018 (6)	−0.0011 (5)
N1	0.0242 (7)	0.0253 (7)	0.0259 (7)	0.0006 (7)	−0.0010 (7)	0.0000 (6)
N2	0.0225 (7)	0.0251 (8)	0.0242 (7)	−0.0003 (6)	0.0009 (6)	0.0010 (6)
C1	0.0318 (11)	0.0275 (9)	0.0329 (10)	−0.0025 (8)	−0.0020 (8)	−0.0001 (8)
C2	0.0354 (11)	0.0292 (10)	0.0402 (11)	−0.0022 (9)	−0.0043 (9)	−0.0074 (8)
C3	0.0365 (11)	0.0404 (11)	0.0302 (10)	−0.0001 (10)	−0.0014 (10)	−0.0109 (8)
C4	0.0296 (10)	0.0363 (10)	0.0273 (9)	0.0006 (8)	−0.0011 (8)	−0.0032 (8)
C5	0.0423 (12)	0.0468 (12)	0.0231 (9)	−0.0016 (10)	0.0031 (8)	−0.0016 (9)
C6	0.0461 (13)	0.0474 (13)	0.0247 (10)	−0.0035 (11)	0.0058 (10)	0.0064 (9)
C7	0.0314 (10)	0.0347 (10)	0.0286 (9)	−0.0023 (9)	0.0038 (8)	0.0045 (8)
C8	0.0386 (12)	0.0341 (11)	0.0348 (11)	−0.0053 (10)	0.0064 (9)	0.0084 (9)
C9	0.0381 (11)	0.0270 (9)	0.0400 (11)	−0.0056 (9)	0.0029 (9)	0.0024 (9)
C10	0.0289 (10)	0.0288 (9)	0.0293 (10)	−0.0023 (8)	0.0015 (8)	−0.0021 (8)
C11	0.0234 (8)	0.0277 (9)	0.0244 (8)	0.0003 (7)	0.0011 (7)	0.0008 (7)
C12	0.0236 (8)	0.0285 (9)	0.0253 (9)	0.0011 (7)	−0.0005 (7)	−0.0006 (7)

### Geometric parameters (Å, º)

**Table:**

Co—O1	2.1386 (13)	C1—C2	1.399 (3)
Co—O1W	2.1110 (14)	C1—H1	0.9500
Co—O2W	2.0782 (15)	C2—C3	1.371 (3)
Co—O3W	2.1024 (14)	C2—H2	0.9500
Co—N1	2.1356 (15)	C3—C4	1.408 (3)
Co—N2	2.1453 (16)	C3—H3	0.9500
S1—O1	1.4997 (13)	C4—C12	1.411 (3)
S1—O2	1.4616 (14)	C4—C5	1.434 (3)
S1—O3	1.4813 (14)	C5—C6	1.351 (4)
S1—O4	1.4784 (14)	C5—H5	0.9500
O1W—H1W	0.837 (12)	C6—C7	1.436 (3)
O1W—H2W	0.834 (13)	C6—H6	0.9500
O2W—H3W	0.840 (13)	C7—C11	1.409 (3)
O2W—H4W	0.853 (12)	C7—C8	1.408 (3)
O3W—H5W	0.822 (12)	C8—C9	1.371 (3)
O3W—H6W	0.822 (13)	C8—H8	0.9500
N1—C1	1.331 (3)	C9—C10	1.397 (3)
N1—C12	1.356 (2)	C9—H9	0.9500
N2—C10	1.329 (3)	C10—H10	0.9500
N2—C11	1.361 (2)	C11—C12	1.441 (3)
			
O2W—Co—O3W	88.05 (6)	N1—C1—C2	122.9 (2)
O2W—Co—O1W	91.48 (6)	N1—C1—H1	118.6
O3W—Co—O1W	86.80 (6)	C2—C1—H1	118.6
O2W—Co—N1	93.13 (6)	C3—C2—C1	119.5 (2)
O3W—Co—N1	99.08 (6)	C3—C2—H2	120.3
O2W—Co—O1	92.59 (6)	C1—C2—H2	120.3
O1—Co—O3W	172.31 (5)	C2—C3—C4	119.26 (19)
O1W—Co—O1	85.53 (5)	C2—C3—H3	120.4
N1—Co—O1	88.54 (6)	C4—C3—H3	120.4
O1W—Co—N1	172.65 (6)	C3—C4—C12	117.42 (19)
O2W—Co—N2	166.55 (6)	C3—C4—C5	123.1 (2)
O3W—Co—N2	83.25 (6)	C12—C4—C5	119.4 (2)
O1W—Co—N2	98.23 (6)	C6—C5—C4	121.0 (2)
N1—Co—N2	78.21 (6)	C6—C5—H5	119.5
O1—Co—N2	97.41 (6)	C4—C5—H5	119.5
O2—S1—O4	110.56 (8)	C5—C6—C7	121.2 (2)
O2—S1—O3	110.35 (8)	C5—C6—H6	119.4
O4—S1—O3	110.04 (8)	C7—C6—H6	119.4
O2—S1—O1	109.85 (8)	C11—C7—C8	117.58 (19)
O4—S1—O1	107.51 (8)	C11—C7—C6	119.2 (2)
O3—S1—O1	108.47 (8)	C8—C7—C6	123.3 (2)
S1—O1—Co	126.85 (8)	C9—C8—C7	119.13 (19)
Co—O1W—H1W	110.3 (18)	C9—C8—H8	120.4
Co—O1W—H2W	128.2 (19)	C7—C8—H8	120.4
H1W—O1W—H2W	93 (3)	C8—C9—C10	119.6 (2)
Co—O2W—H3W	125 (2)	C8—C9—H9	120.2
Co—O2W—H4W	119 (2)	C10—C9—H9	120.2
H3W—O2W—H4W	106 (3)	N2—C10—C9	123.01 (19)
Co—O3W—H5W	117 (2)	N2—C10—H10	118.5
Co—O3W—H6W	132 (2)	C9—C10—H10	118.5
H5W—O3W—H6W	97 (3)	N2—C11—C7	122.72 (18)
C1—N1—C12	118.04 (17)	N2—C11—C12	117.50 (16)
C1—N1—Co	128.58 (14)	C7—C11—C12	119.78 (18)
C12—N1—Co	113.38 (12)	N1—C12—C4	122.85 (18)
C10—N2—C11	117.96 (16)	N1—C12—C11	117.73 (16)
C10—N2—Co	128.85 (14)	C4—C12—C11	119.43 (18)
C11—N2—Co	112.92 (12)		
			
O2—S1—O1—Co	67.05 (11)	C10—N2—C11—C7	−1.0 (3)
O4—S1—O1—Co	−172.59 (9)	Co—N2—C11—C7	−175.48 (16)
O3—S1—O1—Co	−53.64 (11)	C10—N2—C11—C12	179.40 (17)
C12—N1—C1—C2	0.7 (3)	Co—N2—C11—C12	4.9 (2)
Co—N1—C1—C2	179.75 (16)	C8—C7—C11—N2	1.7 (3)
N1—C1—C2—C3	1.7 (3)	C6—C7—C11—N2	−178.4 (2)
C1—C2—C3—C4	−1.6 (3)	C8—C7—C11—C12	−178.70 (19)
C2—C3—C4—C12	−0.7 (3)	C6—C7—C11—C12	1.2 (3)
C2—C3—C4—C5	−178.1 (2)	C1—N1—C12—C4	−3.3 (3)
C3—C4—C5—C6	177.4 (2)	Co—N1—C12—C4	177.58 (15)
C12—C4—C5—C6	0.0 (4)	C1—N1—C12—C11	176.72 (17)
C4—C5—C6—C7	−0.2 (4)	Co—N1—C12—C11	−2.4 (2)
C5—C6—C7—C11	−0.4 (4)	C3—C4—C12—N1	3.2 (3)
C5—C6—C7—C8	179.5 (2)	C5—C4—C12—N1	−179.3 (2)
C11—C7—C8—C9	−1.1 (3)	C3—C4—C12—C11	−176.74 (19)
C6—C7—C8—C9	179.0 (2)	C5—C4—C12—C11	0.8 (3)
C7—C8—C9—C10	−0.1 (4)	N2—C11—C12—N1	−1.7 (3)
C11—N2—C10—C9	−0.3 (3)	C7—C11—C12—N1	178.67 (18)
Co—N2—C10—C9	173.15 (16)	N2—C11—C12—C4	178.26 (17)
C8—C9—C10—N2	0.9 (4)	C7—C11—C12—C4	−1.4 (3)

### Hydrogen-bond geometry (Å, º)

**Table:**

*D*—H···*A*	*D*—H	H···*A*	*D*···*A*	*D*—H···*A*
O1*W*—H1*W*···O3	0.84 (2)	1.89 (2)	2.680 (2)	158 (2)
O1*W*—H2*W*···O1^i^	0.83 (1)	1.95 (1)	2.7818 (19)	172 (2)
O2*W*—H3*W*···O3^ii^	0.84 (2)	1.91 (2)	2.744 (2)	175 (3)
O2*W*—H4*W*···O4^iii^	0.85 (1)	1.93 (1)	2.770 (2)	167 (3)
O3*W*—H5*W*···O4^i^	0.82 (2)	1.95 (2)	2.7548 (19)	168 (3)
O3*W*—H6*W*···O2^iii^	0.82 (2)	1.84 (2)	2.6560 (19)	178 (3)
C3—H3···O2^iv^	0.95	2.45	3.252 (3)	142

Symmetry codes: (i) −*x*+1, *y*−1/2, −*z*+1/2; (ii) −*x*+1, *y*+1/2, −*z*+1/2; (iii) *x*+1, *y*, *z*; (iv) *x*+1/2, −*y*+3/2, −*z*+1.
